# Up and Down States During Slow Oscillations in Slow-Wave Sleep and Different Levels of Anesthesia

**DOI:** 10.3389/fnsys.2021.609645

**Published:** 2021-02-09

**Authors:** Melody Torao-Angosto, Arnau Manasanch, Maurizio Mattia, Maria V. Sanchez-Vives

**Affiliations:** ^1^Institut d’Investigacions Biomediques August Pi i Sunyer (IDIBAPS), Barcelona, Spain; ^2^National Center for Radioprotection and Computational Physics, Istituto Superiore di Sanità, Rome, Italy; ^3^Catalan Institution for Research and Advanced Studies (ICREA), Barcelona, Spain

**Keywords:** Up states, Down states, slow oscillations, sleep, anesthesia, cerebral cortex, cortical model, slow-wave sleep

## Abstract

Slow oscillations are a pattern of synchronized network activity generated by the cerebral cortex. They consist of Up and Down states, which are periods of activity interspersed with periods of silence, respectively. However, even when this is a unique dynamic regime of transitions between Up and Down states, this pattern is not constant: there is a range of oscillatory frequencies (0.1–4 Hz), and the duration of Up vs. Down states during the cycles is variable. This opens many questions. Is there a constant relationship between the duration of Up and Down states? How much do they vary across conditions and oscillatory frequencies? Are there different sub regimes within the slow oscillations? To answer these questions, we aimed to explore a concrete aspect of slow oscillations, Up and Down state durations, across three conditions: deep anesthesia, light anesthesia, and slow-wave sleep (SWS), in the same chronically implanted rats. We found that light anesthesia and SWS have rather similar properties, occupying a small area of the Up and Down state duration space. Deeper levels of anesthesia occupy a larger region of this space, revealing that a large variety of Up and Down state durations can emerge within the slow oscillatory regime. In a network model, we investigated the network parameters that can explain the different points within our bifurcation diagram in which slow oscillations are expressed.

## Introduction

Slow oscillations are an emergent pattern of the cortical network that also recruit subcortical nuclei and, in particular, the cortico-thalamocortical loop. Slow oscillations are the hallmark of slow-wave sleep (SWS), and much research has been carried out to try to understand their role in sleep-induced plasticity and memory consolidation (Diekelmann and Born, 2010; Klinzing et al., 2019). However, this emergent pattern of activity consistent in slow oscillations is generated by the cerebral cortex not only during SWS but also in a variety of pharmacologically induced states (e.g., following administration of propofol, ketamine, urethane, or isoflurane; for a review, see Brown et al., 2010) and also in pathological conditions such as stroke or traumatic brain injury (Sarasso et al., 2019; Cassidy et al., 2020). For this reason, it is important to understand the genesis and dynamics of slow waves (Sanchez-Vives, 2020), as well as the brain state transitions that lead the cerebral cortex in and out of these dynamic regimes.

In this study, we investigated the characteristics of Up and Down states in SWS, deep anesthesia, and lighter anesthesia, in an attempt to generate a “map” of the slow oscillatory space which acts as a common framework for the slow oscillations that are generated under different physiological, pathological, pharmacological, or experimental conditions.

## Materials and Methods

### Surgery and Chronic Implants

All experiments were carried out following Spanish regulatory laws (BOE-A-2013-1337), which comply with European Union guidelines and were supervised and approved by the Animal Experimentation Ethics Committee of the Universitat de Barcelona (287/17 P3).

To obtain local field potential (LFP) long-term recordings in the freely moving rat (Lister-Hooded, 6–10 months old), we carried out chronic implants of electrodes that were inserted 600 microns deep in the cerebral cortex using a stereotaxic apparatus (Kopf Instruments, Tujunga, CA, USA). The recording electrodes were handmade by twisting stainless steel Teflon-insulated wires of 100 μm diameter (California Fine Wire Co., CA, USA). For the recording of the EMG from the neck muscle, a single electrode was made with a 125 μm tungsten-insulated wire (Advent Research Materials Limited, Oxford, UK). The EMG was connected to one of the channels of the preamplifier (Multi Channel Systems, Germany) and acquired at 500 Hz. All signals were amplified ×1000.

The surgery to chronically implant the electrodes was performed with the animal deeply anesthetized with isoflurane (2%). Five to six anchoring stainless steel screws were placed in the skull. A screw located over the cerebellum and connected using a soldered wire was used as ground. The craniotomy was made following the stereotaxic coordinates for the primary visual cortex (V1; −7.3 mm AP, 2.2 mm ML, −0.6 mm DV; Paxinos and Watson, 2007).

After the placement of the electrodes, these were fixed to the skull with an initial application of glue (Loctite 406, Henkel, Germany) and then a second layer of glass ionomer luting cement (Medicaline, Geestland, Germany). Once fixed, the electrodes were welded to the contacts of the case blank connector with crimping contacts (Molex, IL, USA) and in the final step of the surgery, the case was attached to the skull with glass ionomer luting cement. Buprenorphine (0.06 mg/kg) and enrofloxacin (25 mg/kg) were administered for a minimum of 5 days after surgery for analgesia and the prevention and treatment of possible infections. After the post-surgical treatment period, 5 days of handling and habituation to the recording chamber were performed before the initiation of brain recordings, to minimize stress and abrupt movements of the animal during the experimental sessions.

### Recording Protocols

LFPs were recorded from the primary visual cortex of the chronically implanted rats during different anesthesia levels and their natural sleep-wake cycle. For this purpose, the subject was first connected to the headstage preamplifier (Multi Channel Systems, Germany) using a custom-made adapter (IMB-CNM, CSIC) to the implanted case. Then, the animal was placed in a plastic recording cage (57 × 39 × 42 cm), while being videotaped and recorded. The animals were free during all the recordings (not in a stereotaxic). Recordings were acquired, digitized at 5 kHz using a data acquisition interface and Spike 2 software (Cambridge Electronic Design, Cambridge, UK).

### Slow-Wave Sleep Recordings

After the post-surgical recovery period, the animals (*n* = 2) were recorded daily for several hours during their natural sleep-wake cycle of sleep for a minimum of 3 days. SWS periods were classified based on LFP, EMG, and video following the scale for sleep scoring by Iber et al. (2007). Only periods of SWS were included in the current study.

### Anesthesia Recordings

First, a baseline recording was obtained with the awake animal for a minimum of 30 min. Afterward, a mixture of ketamine (Ketolar 50 mg/ml) and medetomidine (Domtor 1 mg/ml) was administered intraperitoneally. The administration of a single subcutaneous injection of atropine immediately after the anesthetic induction (0.05 mg/kg) was part of the anesthetic routine, to reduce bronchial secretions (Sanchez-Vives et al., 2000; Bettinardi et al., 2015; Tort-Colet et al., 2019; Redinbaugh et al., 2020) preventing respiratory obstructions in the deepest phase of the anesthesia. A dose of 0.6 ml of saline was subcutaneously injected every 2 h during the anesthesia for hydration. The rectal temperature was monitored during anesthesia and maintained at 37°C during the recording using a probe and an electric blanket. Two different doses of anesthesia were administered: “light” anesthesia in three animals (20 mg/kg of ketamine and 0.15 mg/kg of medetomidine), and “deep anesthesia” in four animals (40 mg/kg of ketamine and 0.3 mg/kg of medetomidine). Notice that the animals were free during all the recordings (not in a stereotaxic), therefore the anesthesia doses were given only to study the cortical effects and without the need to reach a surgical state of anesthesia. Cortical activity was recorded beginning at injection (induction), throughout the slow oscillatory period of anesthesia until the complete fade-out of the anesthetic effect, all the way to wakefulness.

### Data Analysis

**Figure 1:**
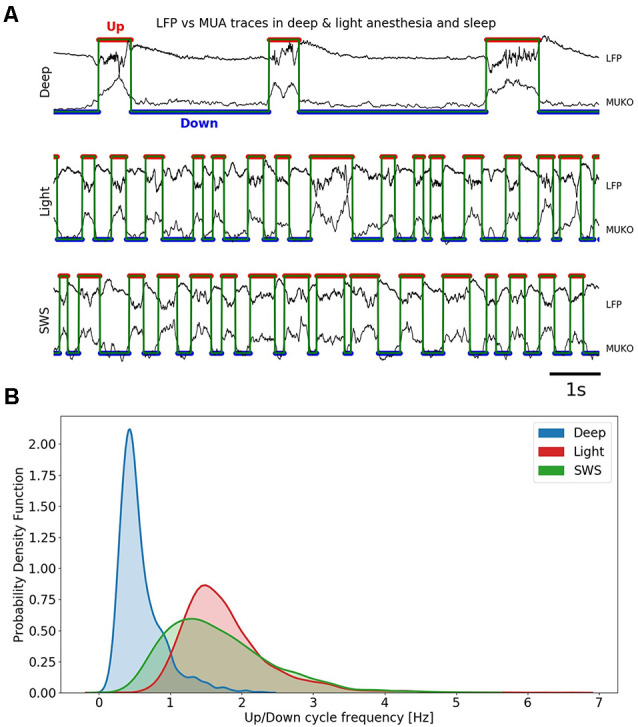
Slow oscillations in different anesthesia levels and slow-wave sleep (SWS).** (A)** Up (red trace) and Down (blue trace) detection shown in local field potential (LFP) and MUA in three different conditions: deep anesthesia, light anesthesia, and SWS. **(B)** Univariate (1D) kernel density estimation (KDE) for the frequency of Up and Down cycles in three different conditions: deep anesthesia, light anesthesia, and SWS.

Raw signals were first downsampled to 3 kHz for computational time reduction purposes. For each recording belonging to an anesthetic condition, the first 10–20 min after a regular establishment of the slow oscillation were selected to compute the Up and Down state duration in the SO cycles. SWS periods in sleep recordings were selected along the entire experiment. Periods of approximately 4 min of SO cycles were extracted per recording. To quantify the durations of Up and Down states, we used the same method as described in Dasilva et al. (2021). Three different characteristics were extracted from the raw signal (LFP) to perform the detection of the Up and Down states (MUKO method, see Figure 1A): the Slow Oscillation deflection (SO), the gamma rhythm, and the firing of the local network (MUA). These characteristics were obtained as time-series: the decimated raw signal, the envelope of the variance of the gamma-filtered signal (Mukovski et al., 2007) and the estimation of the MUA signal from the power of the frequencies in the band between 200 and 1,500 Hz computed in 5 ms windows (Mattia et al., 2010; Reig et al., 2010; Sanchez-Vives et al., 2010; Ruiz-Mejias et al., 2011). To compensate for the high fluctuations in the firing of neurons that are close to the electrode, MUA signal values were logarithmically scaled, thus obtaining the LogMUA signal (Ruiz-Mejias et al., 2011).

**Figure 2:**
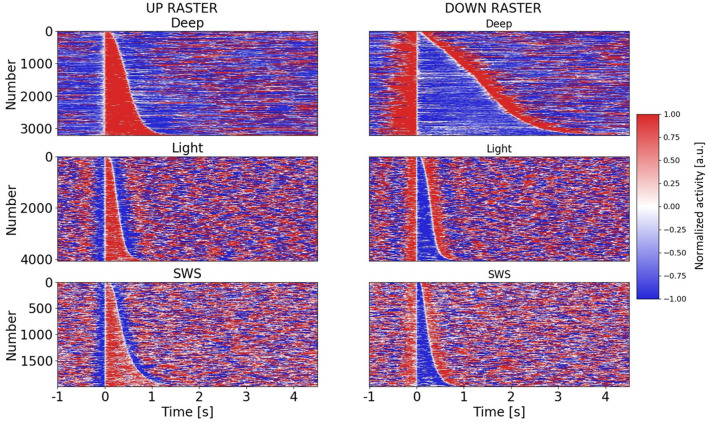
Raster plots of Up and Down states in different anesthesia levels and SWS. Raster plots for all Up and Down states in three different conditions: deep anesthesia, light anesthesia, and SWS. Up and Down states are ordered by duration. For each row in the raster plot, a color-based visualization of the normalized activity (see “Materials and Methods” section) is shown. A 5.5 s (−1 to 4.5 s) window is displayed, with Up or Down states starting at *t* = 0 s.

The multivariate time series composed by the three above signals individually *z*-score normalized were processed relying on a principal component analysis (PCA). Projections on the first principal component resulted in a bimodal distribution, such that the two peaks of the distribution corresponded to samples of network activity belonging to either Up (higher activity) or Down (lower activity) states. Such segregation allowed us to choose a threshold that best separated the Up states from the Down states (Figure 2). To avoid the detection of random fluctuations of the signal that could be detected as the Up States, a minimum duration for the Up and Down durations of 80 ms was set. This threshold was heuristically set after visual inspection of the signal.

For each Up and Down cycle, the duration for both the Up and Down states was computed (Figure 2). Once extracted, they were scattered one against the other to construct the 2D space of points coloring by the condition. Kernel density estimation (KDE) was used to obtain and construct univariate (1D histogram estimate) and bivariate (2D histogram estimate) plots of the Up and Down state durations.

### Simulations

Networks of leaky integrate-and-fire (LIF) neurons (6,880 excitatory and 2,580 inhibitory, E and I, respectively) were simulated relying on an event-based numerical integration (Mattia and Del Giudice, 2000). The network parameters were chosen to model a self-consistent network of Layer 5 neurons each having an average number of pre-synaptic connections computed considering the connection probabilities described in Markram et al. (2015), the cellular densities reported in Beaulieu (1993) and the exponential decay of the cortico-cortical connectivity across layers reported in Schnepel et al. (2015) and in Kätzel et al. (2011) for excitatory and inhibitory pre-synaptic sources, respectively. As result, excitatory neurons received on average 2,910 (129) synaptic contact from E (I) neurons, while the inhibitory ones received 746 (45) connections from E (I) pre-synaptic cells. To model a 1 mm^2^-patch of the cortex only 2% of excitatory connections were considered to be recurrent. Synaptic efficacies were set to have—under mean-field approximation—a fixed point with a firing rate of 0.75 Hz and 4.375 Hz for excitatory and inhibitory neurons (Watson et al., 2016), respectively.

More specifically, for all neurons, the emission threshold was 20 mV and the reset potential was 15 mV, reached by the membrane potential after a refractory period of 2 (1) ms for E (I) neurons following the emission of each spike. Membrane capacitance *C*_m_ was arbitrarily set to 1 leading to express currents in units of mV/s. Decay constant τ_m_ of the membrane potential was 20 and 10 ms for E and I neurons, respectively. Each neuron received as background synaptic noise a Poisson spike train with frequency *v*_ext_ (2,296 Hz and 586.4 Hz for E and I neurons, respectively) transduced in current by instantaneous synaptic transmission with efficacies *J*_ext_ of 0.48 mV and 2.2 mV. The mean external synaptic current was *I*_ext_ = *J*_ext_ *v*_ext_. The probability *c*_αβ_ of having a synapse between pre- and post-synaptic neurons *α* and *β* ϵ {*E*, *I*}, respectively, was {*c*_EE_, *c*_E1_, *c*_1E_, *c*_11_ = {0.6, 5, 0.2, 1.7}%. Synaptic transmission was instantaneous with efficacies {*J*_EE_, *J*_E1_, *J*_1E_, *J*_11_} = {1.9, −1.1, 2.2, −1.1} mV. Spikes from E(I) neurons were delivered with a randomly chosen axonal delay sampled from an exponential distribution with a mean of 22.6 ms (5.7 ms), respectively. All synaptic efficacies were randomly sampled from a Gaussian distribution with mean *J*_aβ_ and a relative standard deviation of 25%. In addition to synaptic currents, each excitatory neuron received an additional potassium current −*g*_a_*a* (*t*) modeling spike-frequency adaptation with strength *g*_a_ and adaptation level $\dot{a}=-\frac{a}{\tau_{a}}+\sum_{k}\delta \left(t-t_{k}\right)$ (Gigante et al., 2007; Mattia and Sanchez-Vives, 2012) having unitary jumps at each spike emission time *t*_k_ of the neuron and decaying with the characteristic time *τ*_a_ = 0.15 s. For each parameter set (*I*_ext_, *g*_a_) we simulated five networks with randomly extracted synaptic coupling for a time span of 100 s.

## Results

To investigate the detailed dynamics of cortical slow oscillations, we studied Up and Down state durations during the slow oscillatory regime *in vivo*. The recordings were obtained from the visual cortex of chronically implanted rats under three conditions: SWS, deep anesthesia, and light anesthesia. The data included here comprises six recording periods in SWS (*n* = 2 rats), five recording periods in deep anesthesia (*n* = 5 rats), and four recording periods in light anesthesia (*n* = 2 rats). In total, 9,271 cycles were analyzed.

The population firing rate was obtained from the recordings of the local field potential (LFP; see “Materials and Methods” section). In Figure 1A, we illustrate traces corresponding to deep anesthesia (top), light anesthesia (middle), and SWS (bottom). The detection of Up and Down states were automatically performed and based on the Slow Oscillation deflection (SO), the gamma rhythm, and the firing of the local network (MUA; see “Materials and Methods” section; MUKO method, Figure 1A); Up and Down states were identified by the red and blue traces, respectively). The frequency of the slow oscillations ranged between 0.1 and 4 Hz, and Figure 1B represents the distribution of frequencies in the three studied conditions. In deep anesthesia, the frequency of oscillation ranged between 0.1 and 2 Hz, but the peak was at 0.45 Hz. In lighter anesthesia, the range of oscillatory frequency was displaced towards higher frequency values, as we have previously described (Tort-Colet et al., 2019; Dasilva et al., 2021). The range of frequencies for light anesthesia was 0.5–3.5 Hz, with a peak at 1.5 Hz. The frequency of slow oscillations in SWS largely overlapped with those in light anesthesia, although expanded towards lower frequencies, ranging between 0.2 and 3.5 Hz, and peaking at 1.3 Hz.

**Figure 3:**
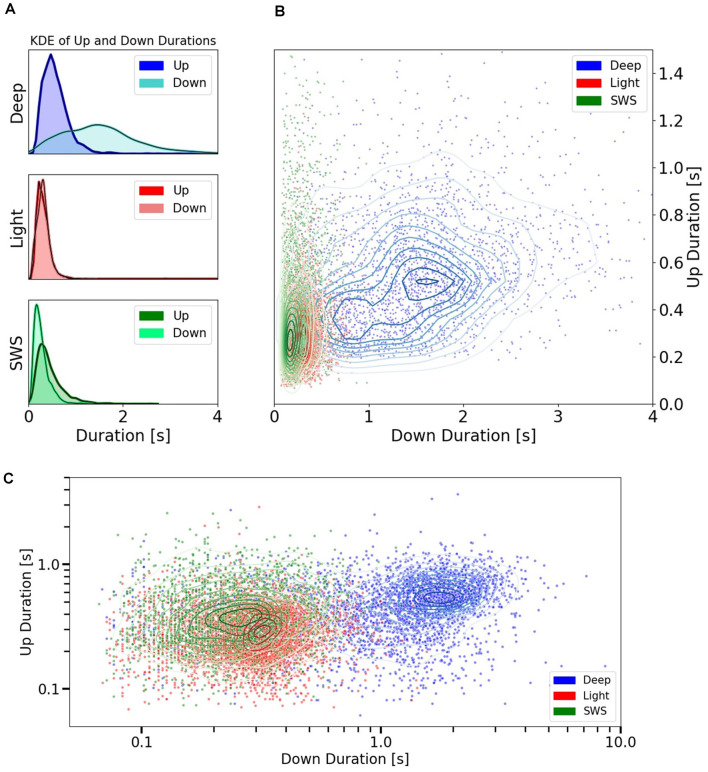
Up and Down state durations in different anesthesia levels and SWS. **(A)** Univariate (1D) KDE (Kernel Density Estimation) for Up and Down state durations in three different conditions: deep anesthesia, light anesthesia, and SWS. **(B)** Scatter plot showing the space of durations for Up and Down cycles. Each point represents the duration of an Up vs. the duration of the subsequent Down. They are colored by conditions: Deep and Light anesthesia or SWS. Irregular ellipses stand for the bivariate (2D) KDE for both Up and Down durations in each of these conditions. **(C)** Scatter plot of Up vs. Down durations in the three conditions (deep and light anesthesia and SWS) represented with a logarithmic scale.

Therefore, including the three experimental conditions, the whole range of frequencies described for slow oscillations (0.1–4 Hz) was covered, opening the door to the investigation of the relationships between Up and Down state durations. The raster plots of the Up and Down states for all the cycles included in this study: 3,205 for deep anesthesia, 4,073 for light anesthesia, and 1,993 for SWS, are displayed in Figure 2. As can be seen in the figure, while the duration of Up (left) and Down (right) states was similar for light anesthesia and SWS, in deep anesthesia both Up and Down states were longer (see exact values next). In particular, Down states could last up to 4 s, and in a few cycles even 9 s (not displayed, see panel Figure 3C). In light anesthesia, the duration of Up and Down states overlapped (Figure 3A), with an average value of 0.31 ± 0.18 s for Up states and 0.31 ± 0.13 s for Down states, therefore the network spent a similar time in the firing periods (Up) and in silence (Down states). However, when anesthesia became deeper, the silent periods became longer, exceeding the periods of firing. The average duration of Up states in deep anesthesia was 0.51 ± 0.33 s, while that of Down states was 1.37 ± 0.83 s (Figure 3A). SWS had similar durations to those in light anesthesia, with an average of 0.45 ± 0.28 s for Up states and 0.28 ± 0.16 s for Down states, with a slight tendency towards longer Down states (Figure 3A). For a statistical evaluation, a Mann-Whitney test comparing Up and Down state durations in the different conditions was performed. A comparison between Light (*n* = 4,073) and Deep (*n* = 3,205) anesthesia showed significantly different Up and Down durations, with a *p* = 0 (given the high number of samples). The same was the case for a comparison between SWS (*n* = 1,993) and Deep (*n* = 3,205) anesthesia, with a *p*-value that was effectively 0 for Up state durations (*p* = 8.8 10^−29^) and *p* = 0 for Down state durations. A comparison between Up and Down durations in Light anesthesia (*n* = 4,073) and SWS (*n* = 1,993) also revealed a significant difference with a *p*-value effectively 0 (*p* = 0 and *p* = 2.6 10^−37^, respectively). Even though the formal statistical test showed that the center of location of each distribution was different—particularly given the large number of samples—the distribution of SWS and light anesthesia presented a large overlap (Figures 3A–C), suggesting that the dynamics in light anesthesia and SWS are fairly similar and different from deeper anesthesia. But what are the characteristics of those dynamics? What is the relationship between Up states and the subsequent Down states?

To explore the relationship between Up and Down states, we represented every cycle with their Up state and subsequent Down state (Figure 3B) to obtain cartography of the space covered by each condition. Deep anesthesia, in blue, covers a large area of the space of relation between Up states and Down states, while the area occupied by light anesthesia and SWS is more restricted, mainly in the sense of never displaying Down states longer than 0.80 s (99th percentile). This data reveals that in these two specific conditions, one physiological (SWS) and one pharmaco-induced (light anesthesia), there was a relatively tight relationship between Up state and Down state duration. However, this is not always the case, and the regime of slow oscillations can be expressed in a larger variety of Up and Down state durations. In Figure 3C, the same data as in Figure 3B is represented using a logarithmic scale, which allows better visualization of the distribution of cycles for SWS and light anesthesia.

**Figure 4:**
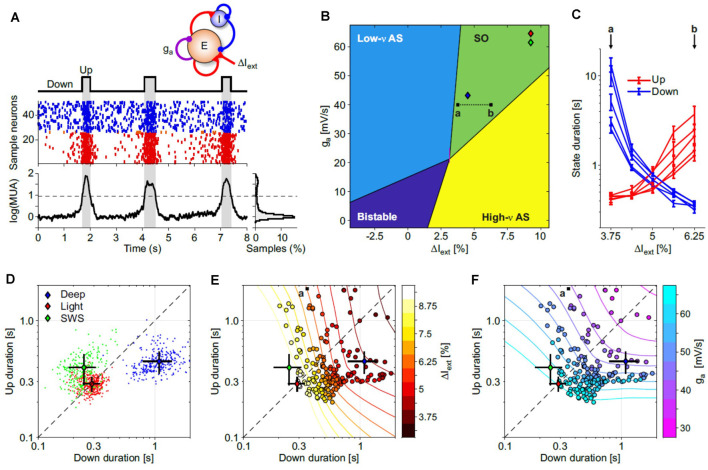
Exploring the Up and Down state durations in *in silico* cortical networks. **(A)** Spontaneous slow oscillations in a network of excitatory and inhibitory leaky integrate-and-fire (LIF) model neurons with spike-frequency adaptation (see “Materials and Methods” section for details). Adaptation is implemented only in excitatory neurons with the strength of self-inhibition *g*_a_ (see top sketch). Middle: emitted spikes by a subset of excitatory (red) and inhibitory (blue) neurons. From the firing rate *v*_E_(*t*) of the excitatory neurons is extracted the multi-unit activity shown at the bottom [log(*MUA* = log(*v*_E_ + 1) + offset, offset is set to have a lower peak of the distribution on the right at 0]. Up and Down states are detected by thresholding log(*MUA*; dashed line). **(B)** Bifurcation diagram of the dynamical regimes expressed by the network model, as in Mattia and Sanchez-Vives (2012). For each point (Δ*I*_ext_*, g*_a_), 100 s of simulations in five independent networks (randomly selected synaptic matrix). Δ*I*_ext_ is the relative change in mean current received by excitatory neurons obtained by changing the external spike rate *v*_ext_. Low-*v* and high-*v* AS (asynchronous state), neuronal spiking is irregular and *v*_E_(*t*) fluctuates around a fixed value at relatively low and high firing rate, respectively. Bistable, the coexistence of both low-*v* and high-*v* AS. SO, the quasi-periodic slow alternation between Up and Down metastable states. Colored diamonds, networks with Up and Down state statistics as in deep and light anesthesia, and sleep experiments (see panel **D**). **(C)** Average Up and Down state durations (red and blue, respectively) computed in five independent networks with parameter changes depicted by the a-b dotted line in panel **(B)**. **(D)** Distributions of average Up and Down durations from experiments under deep (blue) and light (red) anesthesia, and during natural NREM sleep (SWS, green). Colored diamonds, grand-averages (centroids) of the state durations for these three distributions. Black lines, standard deviations of the state durations. **(E,F)** Distribution of average Up and Down state durations from simulated networks (circles) used to work out the diagram in panel **(B)**. Symbol colors represent Δ*I*_ext_ and *g*_a_ in panels **(E,F)**, respectively. Contour lines, isolevel curves of Δ*I*_ext_ and *g*_a_ obtained by fitting with a smoothed surface the mean state durations from simulations. Diamonds as in panel **(D)**. Fitted surfaces were used to infer the effective (Δ*I*_ext_*, g*_a_) needed to reproduce experimental state duration statistics in simulations (diamonds in panel **B**).

What mechanisms can support such a variety of Up and Down state durations? To answer this question, we resorted to the cortical network model similar to Mattia and Sanchez-Vives (2012); (Figure 4A), by varying two parameters: (i) the cortico-cortical synaptic input (Δ*I*_ext_) associated with changes of glutamatergic synaptic transmission; and (ii) the strength of spike-frequency adaptation (*g*_a_) related to activity-dependent hyperpolarizing *K*^+^ currents. These two key features shape the dynamical regime of the model networks giving rise to four different phases (Figure 4B). Two of them are single-attractor asynchronous states, one with high firing (similar to wakefulness) and the other with low firing (e.g., barbiturates, burst suppression-like). In the third bistable regime the network displayed simultaneously two possible stable asynchronous Up- (on) and Down-like (off) states, while in the fourth one, slow oscillations were spontaneously generated. In the parameter subspace where such oscillations were produced, the Up and Down state durations widely varied as shown in Figure 4C, where only the synaptic input changed. This in principle can highlight anticorrelations in the Up and Down state durations (Mattia and Sanchez-Vives, 2012; Levenstein et al., 2019; Nghiem et al., 2020) giving rise to “banana”-shaped distributions at fixed Δ*I*_ext_ or *g*_a_ (Figures 4E,F). Hence, the slow oscillation phase of the cortical network is not an invariant regime; rather, it expresses a wide spectrum of timescales (Mattia and Sanchez-Vives, 2012), which in turn allows us to infer the effective values Δ*I*_ext_ and *g*_a_ needed to reproduce the state duration statistics we measured in different experimental conditions (Figure 4D, as in Figure 3C but averaging chunks of 10 Up-Down cycles). As result, deep anesthesia statistics were produced by networks in the slow oscillation phase close to the boundary with the burst suppression-like regime (Figure 4B, blue diamond): here the Down state was more preferable and as such had longer durations. Light anesthesia and natural SWS (red and green diamonds, respectively) had similar Up/Down duration statistics (Figure 4D) resulting in cortical networks with increased adaptation and cortico-cortical synaptic input. Under this condition the model network was closer to the other boundary separating the SO phase from the awake-like asynchronous state (green and yellow regions, respectively).

## Discussion

In this article, we described slow oscillations as a single dynamic regime that comprises a range of frequencies and a range of Up and Down state durations. Therefore, the regime of slow oscillations is a wide region, which here we investigated with two levels of anesthesia—deep and light—and a physiological condition—SWS. We found that light anesthesia and SWS display similar dynamical features occupying a relatively narrow range of the Up and Down state duration space. Deep anesthesia instead occupies a region of this space displaying a much wider range of possible Up and Down state durations.

All these features can be reproduced in models of cortical networks composed of integrate-and-fire neurons (Gigante et al., 2007; Mattia and Sanchez-Vives, 2012; Jercog et al., 2017). Mean-field approximation and simulations allow us to work out a relatively rich bifurcation diagram for these models relying on two key “forces”: cortico-cortical synaptic strength and adaptation. By varying these two parameters, four spontaneous activity regimes emerge; slow oscillatory regime, bistability, asynchronous state (awake-like), and silent state (coma or barbiturate-like state). In this bifurcation diagram, our light anesthesia and SWS data lie relatively close to the border separating slow oscillations from the awake-like regime. This is a dynamical condition in which microarousals start to emerge (Tort-Colet et al., 2019), interspersed with the periods of coherent slow oscillations on the way to wakefulness. Therefore, light anesthesia, with a tight balance between Up and Down states durations, is a more excitable state which is, therefore, closer to wakefulness. Longer Down states are the expression of a less excitable network, corresponding therefore to a deeper state, further away from wakefulness. This is the case in deep anesthesia, which lies near the border of the state of silence. And in between, a subspace in which different combinations of Up and Down state durations, and thus different frequencies, can be expressed.

Why investigate the parameter space of the slow oscillatory regime? We consider this to be fundamental to understand the multiscale mechanisms that generate these patterns, how the multiple variables in the network move the emergent pattern in different directions or eventually induce a transition towards other global states. Like creating a cartographical map of cortical dynamics, in which we can place the different states induced by physiological (SWS) and pathological states (disorders of consciousness, lesions) or drugs (various anesthetics and doses) that result in slow oscillations, this allows a comparison of results from different studies under the same framework. Furthermore, it is important to interpret the results from different experimental manipulations of slow oscillations (Deco et al., 2009; Sancristóbal et al., 2016; D’Andola et al., 2018) or different species, in a comprehensive dynamical framework.

Our study is not the first to compare SWS and anesthesia. A systematic comparison of intracellular and extracellular patterns during SWS vs. anesthesia was carried out by Chauvette et al. (2011). In this study, it was reported that silent (Down) states were longer under anesthesia than during SWS. These findings are compatible with the data included here since anesthesia induces longer Down states when it is deep, but we also find that light anesthesia induces Down states as short as SWS. Therefore, we find that it is not the anesthesia *per se* that creates different dynamics, but the level of anesthesia, which can result in emergent patterns very similar to SWS.

Interestingly, Nghiem et al. (2020) investigated Up and Down state durations in different species and conditions (SWS, anesthesia) and found a positive correlation between Up and Down state durations while in anesthesia. While in SWS however, Jercog et al. (2017) reported a negative correlation between Up and Down state durations. Our results also show a positive correlation between Up and Down state duration in anesthesia, but we also find a positive correlation in SWS. We previously found a negative correlation between the duration of Up and Down states in cortical slices (Mattia and Sanchez-Vives, 2012), in particular in networks in which inhibition is decreased and spike firing adaptation acquires a prominent role in the emergent dynamics (Sanchez-Vives et al., 2010). This is an interesting illustration of the fact that different mechanisms shape this emergent activity simultaneously, some of which have radically different effects. We propose here that a slight change towards the dominance of short range or long range connections (Bettinardi et al., 2015; Dasilva et al., 2021), or in neuromodulators affecting spike firing adaptation (Barbero-Castillo et al., 2019; Nghiem et al., 2020) can tilt the ongoing slow oscillations in such a way that direct or inverse correlations between Up and Down state durations can be expressed. Indeed, in our network model we find simultaneously a direct correlation due to the simultaneous change of the two key parameters, while within each level an inverse correlation between Up and Down states is embedded (Figures 4E,F).

Slow oscillations have been thoroughly studied for the last quarter of the century [for a review, see Neske (2016), since the characterization made by Steriade et al. (1993)] of an activity known since the first days of EEG. It has been proposed to be the default activity model of the cortical circuit (Sanchez-Vives and Mattia, 2014), that acts as an attractor whenever the cortex becomes structurally or functionally disconnected (Sanchez-Vives et al., 2017) and plays an important role in the functional disruption caused by brain lesions (Sarasso et al., 2019). Still, this apparently simple highly synchronized pattern is difficult to understand in detail and leaves many questions regarding how local and global dynamics in the brain are generated, some of which we have highlighted in this study.

## Data Availability Statement

The datasets presented in this study can be found in Torao-Angosto et al. (2021).

## Ethics Statement

The animal study was reviewed and approved by Animal Experimentation Ethics Committee of the Universitat de Barcelona (287/17 P3).

## Author Contributions

MT-A performed the surgeries and obtained the electrophysiological recordings. AM performed the data analysis. MS-V designed and supervised the study. MM performed the modeling work. All authors contributed to the article and approved the submitted version.

## Conflict of Interest

The authors declare that the research was conducted in the absence of any commercial or financial relationships that could be construed as a potential conflict of interest.

## References

[B1] Barbero-Castillo A. Riefolo F. Matera C. Caldas-Martínez S. Mateos-Aparicio P. Weinert J. F. (2019). Control of brain state transitions with light. bioRxiv [Preprint]. 10.1101/793927 PMC8292914 34018704

[B2] Beaulieu C. (1993). Numerical data on neocortical neurons in adult rat, with special reference to the GABA population. Brain 609, 284–292. 10.1016/0006-8993(93)90884-p 8508310

[B3] Bettinardi R. G. Tort-Colet N. N. Ruiz-Mejias M. Sanchez-Vives M. V. Deco G. (2015). Gradual emergence of spontaneous correlated brain activity during fading of general anesthesia in rats: Evidences from fMRI and local field potentials. NeuroImage 114, 185–198. 10.1016/j.neuroimage.2015.03.037 25804643 PMC4461308

[B4] Brown E. N. Lydic R. Schiff N. D. (2010). General anesthesia, sleep and coma. N. Engl. J. Med. 363, 2638–2650. 10.1056/NEJMra0808281 21190458 PMC3162622

[B5] Cassidy J. M. Wodeyar A. Wu J. Kaur K. Masuda A. K. Srinivasan R.. (2020). Low-frequency oscillations are a biomarker of injury and recovery after stroke. Stroke., 1442–1450. 10.1161/STROKEAHA.120.028932 32299324 PMC7188582

[B6] Chauvette S. Crochet S. Volgushev M. Timofeev I. (2011). Properties of slow oscillation during slow-wave sleep and anesthesia in cats. J. Neurosci. 31, 14998–15008. 10.1523/JNEUROSCI.2339-11.2011 22016533 PMC3209581

[B7] D’Andola M. Weinert J. F. Mattia M. Sanchez-Vives M. V. (2018). Modulation of slow and fast oscillations by direct current stimulation in the cerebral cortex *in vitro*. bioRxiv [Preprint]. 10.1101/246819 PMC12953015 41674091

[B8] Dasilva M. Camassa A. Navarro-Guzman A. Pazienti A. Perez-Mendez L. Zamora-López G.. (2021). Modulation of cortical slow oscillations and complexity across anesthesia levels. NeuroImage 224:117415. 10.1016/j.neuroimage.2020.117415 33011419

[B9] Deco G. Martí D. Ledberg A. Reig R. Sanchez Vives M. V. (2009). Effective reduced diffusion-models: a data driven approach to the analysis of neuronal dynamics. PLoS Comput. Biol. 5:e1000587. 10.1371/journal.pcbi.1000587 19997490 PMC2778141

[B10] Diekelmann S. Born J. (2010). The memory function of sleep. Nat. Rev. Neurosci. 11, 114–126. 10.1038/nrn2762 20046194

[B11] Gigante G. Mattia M. Del Giudice P. (2007). Diverse population-bursting modes of adapting spiking neurons. Phys. Rev. Lett. 98:148101. 10.1103/PhysRevLett.98.148101 17501315

[B12] Iber C. Ancoli-Israel S. Chesson A. L. Quan S. (2007). The AASM Manual for the Scoring of Sleep and Associated Events: Rules, Terminology and Technical Specifications. Westchester, IL: American Academy of Sleep Medicine.

[B13] Jercog D. Roxin A. Barthó P. Luczak A. Compte A. De La Rocha J. (2017). UP-DOWN cortical dynamics reflect state transitions in a bistable network. eLife 6:e22425. 10.7554/eLife.22425 28826485 PMC5582872

[B14] Kätzel D. Zemelman B. V. Buetfering C. Wölfel M. Miesenböck G. (2011). The columnar and laminar organization of inhibitory connections to neocortical excitatory cells. Nat. Neurosci. 14, 100–107. 10.1016/j.jhazmat.2021.125045 21076426 PMC3011044

[B15] Klinzing J. G. Niethard N. Born J. (2019). Mechanisms of systems memory consolidation during sleep. Nat. Neurosci. 22, 1598–1610. 10.1038/s41593-019-0467-3 31451802

[B16] Levenstein D. Buzsáki G. Rinzel J. (2019). NREM sleep in the rodent neocortex and hippocampus reflects excitable dynamics. Nat. Commun. 10:2478. 10.1038/s41467-019-10327-5 31171779 PMC6554409

[B17] Markram H. Muller E. Ramaswamy S. Reimann M. W. Abdellah M. Sanchez C. A.. (2015). Reconstruction and simulation of neocortical microcircuitry. Cell. 163, 456–492. 10.1016/j.cell.2015.09.029 26451489

[B18] Mattia M. Ferraina S. Del Giudice P. (2010). Dissociated multi-unit activity and local field potentials: A theory inspired analysis of a motor decision task. NeuroImage 52, 812–823. 10.1016/j.neuroimage.2010.01.063 20100578

[B19] Mattia M. Del Giudice P. (2000). Efficient event-driven simulation of large networks of spiking neurons and dynamical synapses. Neural Comput. 12, 2305–2329. 10.1162/089976600300014953 11032036

[B20] Mattia M. Sanchez-Vives M. V. (2012). Exploring the spectrum of dynamical regimes and timescales in spontaneous cortical activity. Cogn. Neurodyn. 6, 239–250. 10.1007/s11571-011-9179-4 23730355 PMC3368061

[B21] Mukovski M. Chauvette S. Timofeev I. Volgushev M. (2007). Detection of active and silent states in neocortical neurons from the field potential signal during slow-wave sleep. Cereb. Cortex 17, 400–414. 10.1093/cercor/bhj157 16547348

[B101] Neske G. T. (2016). The slow oscillation in cortical and thalamic networks: mechanisms and functions. Front. Neural Circuits 9:88 10.3389/fncir.2015.00088 26834569 PMC4712264

[B22] Nghiem T.-A. E. A. E. Tort-Colet N. Górski T. Ferrari U. Moghimyfiroozabad S. Goldman J. S.. (2020). Cholinergic switch between two types of slow waves in cerebral cortex. Cereb. Cortex 30, 3451–3466. 10.1093/cercor/bhz320 31989160

[B23] Paxinos G. Watson C. (2007). The Rat Brain in Stereotaxic Coordinates. 6th Edn. New York, NY: Academic Press.

[B24] Redinbaugh M. J. Phillips J. M. Kambi N. A. Mohanta S. Andryk S. Dooley G. L.. (2020). Thalamus modulates consciousness *via* layer-specific control of cortex. Neuron. 106, 66–75. 10.1016/j.neuron.2020.01.005 32053769 PMC7243351

[B25] Reig R. Mattia M. Compte A. Belmonte C. Sanchez-Vives M. V. (2010). Temperature modulation of slow and fast cortical rhythms. J. Neurophysiol. 103, 1253–1261. 10.1152/jn.00890.2009 20032235

[B26] Ruiz-Mejias M. Ciria-Suarez L. Mattia M. Sanchez-Vives M. V. (2011). Slow and fast rhythms generated in the cerebral cortex of the anesthetized mouse. J. Neurophysiol. 106, 2910–2921. 10.1152/jn.00440.2011 21880935

[B27] Sanchez-Vives M. V. Mattia M. Compte A. Perez-Zabalza M. Winograd M. Descalzo V. F.. (2010). Inhibitory modulation of cortical up states. J. Neurophysiol. 104, 1314–1324. 10.1152/jn.00178.2010 20554835

[B28] Sanchez-Vives M. V. Nowak L. G. McCormick D. A. (2000). Membrane mechanisms underlying contrast adaptation in cat area 17 *in vivo*. J. Neurosci. 20, 4267–4285. 10.1523/JNEUROSCI.20-11-04267.2000 10818163 PMC6772627

[B29] Sanchez-Vives M. V. (2020). Origin and dynamics of cortical slow oscillations. Curr. Opin. Physiol. 15, 217–223. 10.1016/j.cophys.2020.04.005

[B30] Sanchez-Vives M. V. Mattia M. (2014). Slow wave activity as the default mode of the cerebral cortex. Arch. Ital. Biol. 152, 147–155. 10.12871/000298292014239 25828686

[B31] Sanchez-Vives M. V. Massimini M. Mattia M. (2017). Shaping the default activity pattern of the cortical network. Neuron 94, 993–1001. 10.1016/j.neuron.2017.05.015 28595056

[B32] Sancristóbal B. Rebollo B. Boada P. Sanchez-Vives M. V. Garcia-Ojalvo J. (2016). Collective stochastic coherence in recurrent neuronal networks. Nat. Phys. 12, 881–887. 10.1038/nphys3739

[B33] Sarasso S. D’Ambrosio S. Fecchio M. Casarotto S. Viganò A. Landi C.. (2019). Local sleep-like cortical reactivity in the awake brain after focal injury. Brain. 143, 3672–3684. 10.1093/brain/awaa338 33188680 PMC7805800

[B34] Schnepel P. Kumar A. Zohar M. Aertsen A. Boucsein C. (2015). Physiology and impact of horizontal connections in rat neocortex. Cereb. Cortex. 25, 3818–3835. 10.1093/cercor/bhu265 25410428

[B35] Steriade M. Contreras D. Curró Dossi R. Nuñez A. Houtkooper R. H. Auwerx J.. (1993). The slow (&lt; 1 Hz) oscillation in reticular thalamic and thalamocortical neurons: scenario of sleep rhythm generation in interacting thalamic and neocortical networks. J. Neurosci. 13, 3284–3299. 10.1523/JNEUROSCI.13-08-03284.1993 8340808 PMC6576531

[B100] Torao-Angosto M. Manasanch A. Mattia M. Sanchez-Vives M. V. (2021). Data associated with “Up and Down states during slow oscillations in slow wave sleep and different levels of anesthesia” [Data set]. Zenodo 10.5281/zenodo.4456700 PMC7900541 33633546

[B36] Tort-Colet N. Capone C. Sanchez-Vives M. V. Mattia M. (2019). Attractor competition enriches cortical dynamics during awakening from anesthesia. bioRxiv [Preprint]. 10.1101/517102 34161772

[B37] Watson B. O. Levenstein D. Greene J. P. Gelinas J. N. Buzsáki G. (2016). Network homeostasis and state dynamics of neocortical sleep. Neuron. 90, 839–852. 10.1016/j.neuron.2016.03.036 27133462 PMC4873379

